# Understanding the seminal plasma proteome and its role in male fertility

**DOI:** 10.1186/s12610-018-0071-5

**Published:** 2018-06-04

**Authors:** Mariana Camargo, Paula Intasqui, Ricardo Pimenta Bertolla

**Affiliations:** 1 Department of Surgery, Division of Urology, Universidade Federal de São Paulo, R Embau, 231, Sao Paulo, SP 04039-060 Brazil; 2 Hospital São Paulo, São Paulo, Brazil

**Keywords:** Male infertility, Proteome, Oxidative stress, Semen analysis, Seminal plasma, Sperm DNA fragmentation, Spinal cord injury, Varicocele, Infertilité masculine, Protéome, Stress oxydatif, Analyse du Sperme, Plasma séminal, Fragmentation de l’ADN spermatique, Blessé médullaire, Varicocèle

## Abstract

Seminal plasma is a complex fluid comprised of secretions from the seminal vesicles, the prostate, bulbourethral glands and from the seminiferous tubule lumen / epididymides / *vasa deferentia*. While it has been established that seminal plasma serves not only as a medium to carry, protect, and nourish sperm after ejaculation up to fertilization, but also as a functional modulator of sperm function, there is still a need to properly characterize the molecular make-up of seminal plasma in fertile men, and to understand how this is altered in different causes of male infertility. The main purpose of this manuscript was to review articles that studied the human seminal plasma proteome, ranging from characterizing a fertile seminal plasma proteomic map to studies comparing seminal plasma from fertile and infertile men, and comparing seminal plasma of fertile or normozoospermic men to a diverse range of biological causes for male infertility. Finally, this review has focused on the association between semen and sperm functional quality and the seminal plasma proteome, in order to demonstrate cellular and molecular mechanisms of male infertility. Due to the untargeted nature of the majority of the studies presented in this review, and to the diverse range of techniques utilized to study the seminal plasma proteomic composition, many differentially expressed proteins were observed. However, in general, it seems that there is a seminal plasma proteome associated to male fertility, and that different biological conditions or cellular phenotypes shift its pathways away from its homeostatic condition to altered energy production pathways. Moreover, it seems there is an inflammatory component to the seminal plasma of infertile men. In conclusion, there are a number of studies focused on the proteomic composition of human seminal plasma; downstream confirmatory studies will help to understand specific pathways of infertility in different biological conditions.

## Résumé

Le plasma séminal est un liquide complexe comprenant les sécrétions des vésicules séminales, de la prostate, des glandes bulbo-urétrales, et des sécrétions provenant de la lumière des tubes séminifères/épididymes/canaux déférents. Bien qu’il a été établi que le plasma séminal n’est pas seulement un milieu servant à transporter, protéger et nourrir les spermatozoïdes après l’éjaculation et jusqu’à la fécondation, mais qu’il constitue aussi un modulateur fonctionnel des fonctions spermatiques, il demeure nécessaire de caractiser de manière appropriée la constitution moléculaire du plasma séminal des hommes féconds, et de comprendre comment celle-ci est altérée dans les différentes causes d’infertilité masculine.

Le principal objectif de cet article est de passer en revue les études du protéome du plasma séminal, en allant de celles ayant caractérisé une carte protéomique du plasma séminal fertile aux études ayant comparé le plasma séminal d’hommes féconds et inféconds et à celles ayant comparé le plasma séminal d’hommes féconds ou normozoospermiques à celui d’hommes présentant diverses causes d’infertilité. Pour finir, la présente revue est centrée sur l’association entre d’une part la qualité fonctionnelle du sperme et des spermatozoïdes et d’autre part le protéome du plasma séminal dans le but de démontrer les mécanismes cellulaires et moléculaires de l’infertilité masculine. En raison de la nature non ciblée de la majorité des études présentées dans cette revue, et de la grande diversité des techniques utilisées pour étudier la composition protéomique du plasma séminal, de nombreuses protéines différentiellement exprimées ont été observées.

Cependant, d’une façon globale, il semblerait qu’il y ait un protéome séminal associé à la fertilité masculine et que des situations biologiques ou des phénotypes cellulaires particuliers l’éloignerait de son point d’équilibre vers des états associés à une production énergétique altérée. De plus, il semblerait exister une composante inflammatoire du plasma séminal chez les hommes infertiles. En conclusion, il existe de nombreuses études centrées sur la composition protéomique du plasma séminal humain; de futures études de confirmation seront utiles à la compréhension des voies spécifiques de l’infertilité dans ses différentes conditions biologiques.

## Background

Infertility is defined as the inability to achieve pregnancy after 12 months of unprotected and regularly distributed within the menstrual cycle, sexual intercourse [1]. Initial screening for infertility in the male counterpart should include: (i) physical examination [2] including scrotal palpation [3], and (ii) at least one semen analysis [1]. However, it is noteworthy that these techniques are unable to diagnose the male fertile potential or testicular dysfunction, especially because semen analysis can be normal in infertile men. Moreover, semen analysis can be abnormal even without any diagnosed cause of male infertility. Thus, semen analysis has a limited predictive value [4].

Sperm functional evaluation, associated with semen analysis, have been studied in the recent past in order to understand the sperm traits important for fertilization, as well as the level of testicular damage [5]. These tests are performed in order to increase sensibility in detecting alterations that are associated to male infertility, and that function as surrogate end-points for male infertility studies [6], as well as to determine medical conduct during assisted reproduction treatments [4]. Examples of such tests are analysis of mitochondrial activity, acrosome integrity, semen oxidative stress, sperm penetration assay, and sperm DNA fragmentation [4, 7–10]. However, while different studies associate high rates of DNA fragmentation to low rates in vitro fertilization (IVF) success [11, 12] and to recurrent miscarriage [13], the American Society for Reproductive Medicine states that there are insufficient data to recommend routine testing of DNA fragmentation [1].

Moreover, sperm functional alterations indicate a testicular damage that has already established itself. Early detection of testicular damage is warranted if one is to intervene before there is testicular damage reflected in semen quality. With this in mind, in recent years, seminal plasma proteomics analysis has aided expansion and improvement of clinical diagnostic testing for infertility [14]. However, as is usually the case with so-called hot topics in research, a diverse range of articles has been produced, including different techniques, end-points, quality controls, and confirmation. How much this has critically led current knowledge of the molecular mechanisms of male infertility remains to be answered. Thus, this review aimed to analyze the published literature on the seminal plasma proteome, and to verify if it is able to differentiate high and low fertile potential.

## Rationale

The rationale that supports studying the seminal plasma proteome in search for the explanation – and eventually diagnosis – of male infertility is based on at least four statements previously demonstrated in the literature:

- *seminal plasma is functional*: two elegant studies performed by Henault and Killian and by Brackett et al. [15, 16] demonstrated that seminal plasma is functional and essential for sperm survival and fertilization. Henault and Killian demonstrated that addition of seminal plasma from sires with high fertility to sperm from sires of low fertility increased their ability to penetrate zona-free oocytes. The opposite (decrease of fertility by adding seminal plasma of low fertility sires to sperm from high fertility sires) was also observed. In a similar manner, Brackett et al. mixed seminal plasma of spinal cord injured (SCI) men with sperm from healthy controls, and verified a decrease in sperm motility. Conversely, when seminal plasma of healthy men was added to sperm of SCI men, an increase in sperm motility was observed [17].
- *Accessory sex gland proteins bind to the sperm membrane and affect function:* the removal of accessory sex glands in golden hamsters (*Mesocricetus auratus*) decrease implantation rates, alters embryo development, and increase embryo loss [18–20]. These studies demonstrated that seminal plasma plays an essential role in sperm survival and fertilization.
- *seminal vesicle fluid proteins have been shown to relate to fertility*: in bovines, Moura et al. [21] studied the proteome of accessory sex gland fluid from sires of high and low fertility – divided according to their known fertility indexes. The authors observed overexpression of seminal vesicle proteins, such as spermadhesins in low fertility sires and osteopontin in high fertility sires. Both proteins are produced in the seminal vesicles and have a direct effect on sperm function during fertilization [21, 22]. In a follow-up study, the authors verified that these proteins interact with the sperm membrane during capacitation and assist penetration of the oocyte [23].
- *fluid of testicular/epididymal origin contributes to the seminal plasma proteome in a detectable (and quantifiable) manner:* the total ejaculate volume is originated from the seminal vesicles (65% of the ejaculate volume), the prostate (25% of the ejaculate volume) and the testes and epididymides (10% of the ejaculate volume) [14]. However, even with the relatively low contribution of epididymal/deferential fluid to the overall ejaculate volume, proteomic analysis of seminal plasma of healthy men versus post-vasectomized men demonstrated that almost 12% of seminal plasma proteins are of testicular/epididymal/deferential origin (280 proteins from 2360 found in total) [14]. The authors concluded that there are post-ejaculate proteins in the testes and epididymides that affect sperm function after ejaculation.

Seminal plasma is the liquid component of semen, that nurtures them during their transit in the female reproductive tract [24]. Seminal plasma proteins have the ability to interact with different molecules in order to respond to, and modulate, their milieu during sperm capacitation and during sperm-egg interaction [25, 26]. In order to effect these responses, seminal plasma is of a complex nature, especially because it arises from different organs or tissues [27] and controls a diverse range of mechanisms, including triggers for sperm capacitation and interaction with the surrounding secretions in the female reproductive tract [6]. Therefore, due to its functional nature, it has been proposed that using the seminal plasma to predict fertility is promising [6].

## Review criteria

A literature search was performed using PubMed and Google scholar electronic databases, with the following keywords: “seminal plasma proteomic”, “seminal plasma proteome”, “seminal plasma AND proteome”, “seminal plasma AND proteomic”, “male seminal plasma proteome characterization”, “male seminal plasma proteomic profile”, “varicocele AND seminal plasma proteome”, “varicocele AND seminal plasma proteomic”, “seminal plasma proteome AND obesity”, “seminal plasma proteomic AND obesity”, “smoking AND seminal plasma proteomic”, “smoking AND seminal plasma proteome”, “spinal cord injury AND seminal plasma proteome”, “spinal cord injury AND seminal plasma proteome”, “seminal plasma proteome AND semen analysis”, “seminal plasma proteomic AND semen analysis”, “seminal plasma proteome AND oligozoospermia”, “seminal plasma proteome AND teratozoospermia”, “seminal plasma proteome AND asthenozoospermia”, “seminal plasma proteome AND sperm function”, “seminal plasma proteome AND sperm DNA fragmentation”, “seminal plasma proteome AND mitochondria”, “seminal plasma proteome AND acrosome”, and “seminal plasma proteome AND oxidative stress”. Only articles written in English in peer reviewed journal were selected. The articles selected were published until November 2017.

## Seminal plasma proteins as markers of fertility

The human seminal plasma proteome has been studied since 1984 [28], when Rui et al. observed the ejaculate by dividing it in different fractions. In that study, the authors verified different proteins from the prostate fraction and the seminal vesicle fraction using 2D gel electrophoresis (2DGE). By that time, the authors could already verify a large number of low molecular mass proteins in the vesicular fraction, and the cellular contribution from the testis and epididymis [28].

### Characterizing the seminal plasma proteome of fertile men

Two studies proposed to characterize the seminal plasma proteome, as a means of producing a general overview of the identified seminal plasma proteins, without any focus on determining infertility or biological state. Initially, Pilch and Mann published the first study with a large amount of proteins observed in seminal plasma [24]. The authors performed 2D electrophoresis followed by liquid chromatography mass spectrometry (LC MS/MS) and, 923 proteins were found. In that study, 90% of the identified proteins had never been previously described in the male reproductive tract, and thus, the authors were able to demonstrate the complexity of the seminal plasma proteome [24]. Proteins were mainly from seminal vesicles (Fibronectin (FN1), Semenogelin-1 (SEMG1) and Semenogelin-2 (SEMG2)). Lactotransferrin (LTF) and all three chains of heterotrimeric laminin were also abundant in seminal plasma [24]. Milardi et al. then evaluated the seminal plasma of 5 men from couples who had recently achieved pregnancy [29]. The authors observed around 1000 proteins, but only 83 were common to the 5 men analyzed, including SEMG1, SEMG2, LTF, Olfactory receptor 5R1 (OR5R1), Clusterin (CLU), and E3 ubiquitin-protein ligase UBR5 (UBR5) [29]. In both studies above, these proteins observed were mostly involved in protein binding (~ 50%) and catalytic activity (~ 39%), and were mainly cytoplasmic (58.7%, such as proteins found in vesicles and the endomembrane system or in the cytoskeleton) or extracellular (21.2%). The authors concluded that seminal plasma lacks nucleic acid-binding proteins, transcription regulators, and membrane receptors and channels [24, 29].

### Men of known fertility versus men with altered spermatogenesis

Aiming to determine biomarkers for fertility, other studies have compared the seminal plasma proteome of fertile and infertile men. However, the classification of fertile and infertile men in each study is different, which adds a potential bias to this analysis. A full complete list of suggested biomarkers for semen alterations are present in Table 1. These proteins were assigned tissue of expression based on their location in the Human Protein Atlas [30] and Genecards [31] databases, and were grouped as: (i) proteins expressed exclusively in the testes and epididymis, (ii) proteins expressed in all different male tissues, including the testes, and (iii) proteins not produced in testes but expressed in other reproductive tissues.

**Table 1:** Suggested biomarkers for different infertility factors. Tissue expression data was collected from the Human Protein Atlas [30] and Genecards [31] databases

Gene name	Protein name	Infertility factor	Expression	References
Expressed in testes/epididymis but not in other male reproductive tissues				
ALB	Albumin	Oxidative stress	Increased	[50]
CRISP1	Cysteine-rich secretory protein 1	Non-obstructive azoospermia	Increased	[42]
EDDM3A	Epididymal secretory protein E3-alpha	Sperm DNA fragmentation	Increased	[48]
FTMT	Mitochondrial ferritin	Sperm DNA fragmentation	Decreased	[49]
GAPDHS	Glyceraldehyde-3-phosphate dehydrogenase testis specific	Obstructive azoospermia	Absent	[14]
HIST1H2BA	Histone H2B type 1-A	Obstructive azoospermia	Absent	[14]
LDHC	L-lactate dehydrogenase C chain	OAT	Absent	[40]
PGK2	Phosphoglycerate kinase 2	Obstructive azoospermia	Absent	[14]
TEX101	Testis-expressed sequence 101	Obstructive azoospermia	Absent	[14, 38–40]
TKTL1	Transketolase-like protein 1	NOA, OA	Absent	[40]
Expressed in different male reproductive tissues				
ANXA7	Annexin-7	Low mitochondrial activity	Increased	[8]
CEP135	Centrosomal protein of 135 kDa	Non-obstructive azoospermia	Absent	[36]
CYCS	Cytochrome C	Asthenozoospermic	Increased	[49]
CLU	Clusterin isoform-1	Oligozoospermic	Decreased	[47]
COL6A2	Collagen alpha-2(VI) chain	Non-obstructive azoospermia	Exclusive	[41]
DJ1	Protein/nucleic acid deglycase DJ-1	Asthenozoospermia	Decreased	[44]
ERP44	Endoplasmic reticulum resident protein 44	Low mitochondrial activity	Increased	[8]
FN1	Fibronectin I isoform 3 preprotein	Oxidative stress	Decreased	[50]
GGT7	Gamma-glutamyltransferase 7	Non-obstructive azoospermia	Exclusive	[41]
GSTM3	Glutathione S-transferase Mu3	Low mitochondrial activity	Increased	[8]
LCN1	Lipocalin-1	OAT	Increased	[46]
LGALS3BP	Galectin 3 binding	Oxidative stress	Decreased	[50]
M2BP	Galectin-3-binding protein	OAT	Decreased	[46]
MIF	Macrophage migration inhibitory factor-1 peptide	Oxidative stress	Decreased	[50]
NPC2	Epididymal secretory protein E1	Obstructive azoospermia	Absent	[36]
NPC2	Epididymal secretory protein E1	OAT	Decreased	[46]
PLTP	Phospholipid transfer protein	Low acrosome integrity	Increased	[8]
PSMB5	Proteasome subunit alpha type-5	Sperm DNA fragmentation	Increased	[8]
RASGRF1	Raf-specific guanine nucleotide-releasing factor 1	Non-obstructive azoospermia	Absent	[36]
RNASE4	Ribonuclease 4	Sperm DNA fragmentation	Increased	[48]
SORD	Sorbitol dehydrogenase	Non-obstructive azoospermia	Exclusive	[41]
Not expressed in the testes but expressed in the epididymis or other male reproductive tissues				
AZGP1	Zinc alpha-2-glycoprotein	Oligozoospermic	Increased	[47]
CST4	Cystatin S precursor	Oxidative stress	Increased	[50]
KLK3	Kallikrein-3	Oxidative stress	Increased	[50]
LTF	Lactotransferrin	Oxidative stress	Increased	[50]
MUC5B	Mucin-5B	Oxidative stress	Increased	[9]
PIP	Prolactin inducible protein	Non-obstructive azoospermia	Absent	[36]
PIP	Prolactin inducible protein	OAT	Increased	[46, 47]
STAB2	Stabilin-2	Non-obstructive azoospermia	Absent	[36]

*OAT Oligoasthenoteratozoospermia

The first comparison of the human seminal plasma proteome between fertile and infertile men was performed in 2001, by Starita-Geribaldi et al. [32]. The proteomic profile of 5 fertile men, 4 vasectomized men, and 4 azoospermic men was studied. The authors performed 2DGE, followed by Matrix-assisted laser desorption/ionization time of flight mass spectrometry (MALDI-TOF) for spot identification. 2DGE allowed the detection of 750 spots in the seminal plasma of fertile men. However, the authors did not identify any of the spots [32].

Other authors have focused on studying the seminal plasma proteome of azoospermic men, in order to determine biomarkers for obstructive (OA) and non-obstructive azoospermia (NOA) [33–35]. Yamakawa et al. analyzed the seminal plasma proteome of 10 fertile men and 10 infertile azoospermic men (7 NOA and 3 OA patients) [36]. The authors showed that Epididymal secretory protein E1 (NPC2) was absent in OA, but not in NOA, whereas 4 proteins (Prolactin-inducible protein - PIP, Stabilin-2 – STAB2, Centrosomal protein of 135 kDa – CEP135, and Ras-specific guanine nucleotide-releasing factor 1 – RASGRF1) were absent in more than 3 patients with NOA. These proteins were suggested as biomarkers of each infertility condition. Other proteins were also absent in NOA and OA patients: Transketolase-like protein 1 (TKTL1), L-lactate dehydrogenase C chain (LDHC) and PGK2 [37]. Furthermore, Batruch et al. compared seminal plasma proteome of fertile men (*n* = 5) and vasectomized men (*n* = 5). The samples were analyzed by strong-cation exchange LC MS/MS. The authors verified exclusive proteins from the testes and epididymis, such as: Testis-expressed sequence 101 protein (TEX101), Phosphoglycerate kinase 2 (PGK2), Histone H2B type 1-A (HIST1H2BA), and Glyceraldehyde-3-phosphate dehydrogenase testis-specific (GAPDHS). These proteins have important reproductive functions and may be potential biomarkers of obstructive azoospermia [14]. On the other hand, different studies confirmed that TEX101 protein in different levels can be diagnostic of male infertility, including NOA and OA [38–40].

Batruch et al. studied 5 men with NOA and compared them with results from the previous study. In total, 18 proteins were exclusively expressed in NOA, such as: Collagen alpha-2 (VI) chain (COL6A2, previously identified overexpressed in post-vasectomy samples), Gamma-glutamyltransferase 7(GGT7), and Sorbitol dehydrogenase (SORD). Fructose metabolism was enriched in this group, indicating hypospermatogenesis or maturation arrest, according to the authors [41]. In another study, Cysteine-rich secretory protein 1 (CRISP1) was able to differentiate NOA from OA [42] . In sum, finding a seminal biomarker that is able to differentiate NOA from OA is necessary, and constitutes a promising field.

Cadavid et al. studied men with proven fertility and compared them to men with infertility (defined by over 1 year of attempt without contraceptive measures). Seminal plasma proteomics analysis was performed by surface-enhanced laser desorption/ionization-time of flight mass spectrometry (SELDI-TOF-MS), in which proteins of certain affinities are bound to a target plate for downstream identification. Their results demonstrated 10 over-expressed proteins in the infertile group, including: Ubiquitin-conjugating enzyme E2C binding protein (UBE2C), Cystatin-A (CSTA), Dermcidin (DCD), Ceruloplasmin (CP), Ras GTPase-activating-like protein IQGAP1 (IQGAP1). The authors suggest that these proteins could be candidates for biomarkers in male infertility [43].

Wang et al. compared seminal plasma proteins of healthy control donors to asthenozoospermic men, using LC-MS/MS. A total of 741 proteins were identified [44], of which 45 were increased and 56 were decreased in asthernozoospermic men. Most of the proteins originated from the prostate and epididymis, and presented catalytic activities, being Protein/nucleic acid deglycase DJ-1 protein (DJ1) the most down-regulated protein in asthenozoospermic group [44]. Similarly, Herwig et al. compared the seminal plasma proteome of fertile patients to idiophatic oligoasthenoteratozoospermic (iOAT) men by LC-MS/MS [45]. A total of 2489 proteins were identified, of which 505 proteins exclusively observed in iOAT men, when compared to fertile men, 744 absent in iOAT, and 24 overexpressed in iOAT patients. The proteins were mostly involved with response to stress, system development, and anatomical structure development functions [45]. Giancomini et al. also compared 10 normozoospermic men to OAT patients, using 2DGE followed by MS for protein identification. Six different spots were of different intensity levels, and were therefore selected for MS analysis. Of these, 4 proteins were identified: Epidydimal secretory protein E1 (NPC) and Galectin-3-binding protein (M2BP) lower in the OAT group, and Lipocalin-1 (LCN1) and Prolactin inducible protein (PIP), higher in the OAT group [46].

Sharma et al. studied the impairment of spermatogenesis in a different manner, by recruiting 12 patients and dividing then according to their seminal alteration (normozoospermic, oligozoospermic, teratozoospermic and oligoteratozoospermic). Their results demonstrated 20 proteins differentially expressed between the 4 groups [47], of which Clusterin isoform 1 (CLU) was decreased and Zinc alpha-2-glycoprotein (AZGP1) was increased in oligospermic patients. The proteins differentially expressed in this study agree with the results of Cadavid et al. and Wang et al. [43, 44, 47].

### Men of known fertility versus men with altered sperm function and seminal oxidative stress

The seminal plasma proteome has been shown to reflect spermatogenesis and epididymal sperm maturation [8], and many proteins in seminal plasma are of testicular or epididymal origin [14]. These observations have paved the way for studies that have sought to observe and understand the relation between the seminal plasma molecular composition and its corresponding cellular phenotype. Not only has this brought information regarding mechanisms of male infertility, but it has also suggested protein targets for future clinical intervention [8, 9, 48–50].

Regarding sperm functional alterations, 3 different studies verified the seminal plasma proteomic profile of men with high sperm DNA fragmentation [8, 48, 49]. Behrouzi et al. compared 24 normozoospermic men with 34 men with altered semen analysis and/or sperm DNA damage using 1DGE followed by LC-MS/MS [49]. In controls, the authors observed increased levels of proteins involved in mitochondrial function, of which Mitochondrial ferritin (FTMT) was the most prominent. In addition, Cytochrome C (CYCS) was observed only in patients with low sperm motility but normal DNA fragmentation. For high sperm DNA fragmentation patients, no exclusive proteins were observed; however, proteins related to DNA binding and some histone proteins were observed overexpressed in these patients [49].

In an initial study, Intasqui et al. ranked 89 normozoospermic men according to their sperm DNA fragmentation levels, and utilized samples from the highest and lowest ranked patients for 2D nanoUPLC-ESI-MS^E^ shotgun proteomics analysis [48]. Eighteen samples with low sperm DNA fragmentation and 18 men with high sperm DNA fragmentation were selected. Proteomics results demonstrated 72 proteins differentially expressed between the groups, of which 21 proteins were increased in the high sperm DNA fragmentation samples. Among them, Epididymal secretory protein E3-alpha (EDDM3A) and Ribonuclease 4 (RNASE4), both of which participate in endoribonuclease activity, were observed. In a follow-up study, the same authors studied the seminal plasma proteomic profile of men with high versus low sperm DNA fragmentation, low versus high acrosome integrity, and low versus high mitochondrial activity [8]. For this follow-up study, 156 normozoospermic patients were recruited and ranked according to their sperm functional analysis results (DNA fragmentation, acrosome integrity or mitochondrial activity). In total, 40 proteins were decreased and 64 increased in patients with low mitochondrial activity. Some proteins were suggested as potential biomarkers for sperm mitochondrial activity alterations because they were also significant utilizing multivariate statistical analysis tests: Annexin-7 (ANXA7), Glutathione S-transferase Mu3 (GSTM3), and Endoplasmic reticulum resident protein 44 (ERP44). These proteins are involved in acrosome reaction, mitochondrial integrity and oxidative stress protection. Regarding acrosome integrity studies, 27 proteins were decreased and 49 increased in the low acrosome integrity samples. Of these, only one protein was cross-validated in their multivariate statistical analysis: Phospholipid transfer protein (PLTP), a protein associated with acute phase response [8]. With regards to sperm DNA fragmentation 108 proteins were decreased and 26 were increased in the high sperm DNA fragmentation group. A single protein suggested as biomarkers for high sperm DNA fragmentation was: proteasome subunit alpha type-5 (PSMB5) [8].

Two different studies performed seminal plasma proteomic profile analysis in seminal plasma of men with high levels of oxidative stress, in order to verify how it affects seminal plasma proteins [9, 50]. In 2013, Sharma et al. evaluated oxidative stress and total antioxidant capacitation of 20 healthy male volunteers and 32 infertile men. The infertile patients were categorized into Reactive Oxygen Species (ROS) positive or ROS negative, and then the infertile and fertile patients were pooled into three different groups and submitted to LC-MS/MS. Proteomics analysis showed 14 proteins, of which 7 were identified in both ROS positive and ROS negative groups, 3 proteins were identified only in the ROS negative group (FN1, Macrophage migration inhibitory factor-1 peptide (MIF) and Galectin 3 binding (LGALS3BP), and 4 proteins were uniquely expressed in the ROS positive group: Cystatin S precursor (CST4), Albumin (ALB), LTF and KLK3.

Intasqui et al. prospectively analyzed semen oxidative stress levels (measured as lipid peroxidation levels) in 156 normozoospermic men [9]. The authors then ranked patients by lipid peroxidation levels, and included 23 men with highest levels as a “high oxidative stress” group, and 23 men with lowest levels as a “low oxidative stress” group. LC-MS/MS proteomics experiments were performed, and 629 proteins were identified in the study, of which 23 were lower and 71 were higher in seminal plasma of patients with high lipid peroxidation levels. Gene ontology and Kyoto Encyclopedia of Genes and Genomes (KEGG) functional enrichment analysis demonstrated unsaturated fatty acids biosynthesis, antioxidants and oxidants activity, cellular response to heat stress and immune response. One protein was also suggested as a potential seminal biomarker of oxidative stress: Mucin-5B (MUC5B) [9]. Therefore, while in the study by Sharma et al. the authors suggest potential biomarkers for oxidative stress associated to infertility [50], Intasqui et al. suggested a biomarker for the verification of oxidative stress in normozoospermic patients, which the authors discuss could be an early measure for oxidative stress [9].

### Different biological conditions

Another promising field of seminal plasma proteomics studies in male infertility is the study of how different biological conditions – such as the presence of hypogonadism, varicocele, or anejaculation due to spinal cord injuries, for example, affect the seminal proteome [51–58]. These studies have the potential not only to elucidate the molecular mechanisms underlying a certain disease, but also to differentiate each biological condition, thus offering a comprehension of the mechanisms of the disease, a prognostic capability, and a diagnostic potential. In varicocele, for example, seminal plasma proteomics analysis has been used to differentiate a deleterious phenotype from a “silent” varicocele [5, 58]. A complete list of seminal plasma studies regarding different biological conditions, and their respective suggested biomarkers are present in Table 2.

**Table 2:** Studies of the seminal plasma proteomic profile in different biological conditions

Author	Biological condition	Study design	Identified and differentially expressed proteins	Main expressed proteins	Main molecular functions
Zylbersztejn et al. 2013 [56]	Varicocele	- 67 adolescents in total: - 21 without varicocele and normal SA; - 28 with varicocele and normal SA; - 18 with varicocele and abnormal SA.	From 181 to 323 spots per gel: - 31 proteins in adolescent without varicocele; - 7 proteins in adolescent with varicocele and normal SA; - 11 proteins in adolescents with varicocele and abnormal SA	- Adolescent without varicocele: SEMG1, PIP and NPC2 - Adolescent with varicocele and normal SA: EP3B, SEMG2, SAP and BRE1B - Adolescent with varicocele and abnormal SA: CYTS, PPAP and IBP3.	- Spermatogenesis - Transport of molecules - Immune response - Inflammatory pathways - Apoptosis
Del Giudice et al. 2013 [59]	Varicocele	19 adolescents before and after varicocelectomy	From 84 to 168 spots per gel: - 4 proteins overexpressed in pre-varicocelectomy adolescents -	- Overexpressed in Pre: Albumin, POLR3B, PIP and NELFE - Exclusive in Post: SEMG2, SEMG1, PIP, PSA, TGM4, SERPINA1	- Sperm-binding - Metal-iron-binding - Innate immune response
Camargo et al. 2013 [55]	Varicocele	18 adult men before and after varicocelectomy	- 316 proteins identified in total: - 58 proteins overexpressed or exclusive in pre-varicocelectomy - 38 proteins exclusive in post-varicocelectomy	- Pre-varicocelectomy: ANXA5, LDHA, CLU, HSP90AB1 and HSP90AA1 - Post-varicocelectomy: DJ1, SOD1, S100A9, ANXA1, GAPDH and MDH1.	- Cellular response to ROS - NAD-binding - Nitric oxide metabolic process - TPR domain - Eicosanoid biosynthetic process
Del Giudice et al. 2016 [57]	Varicocele	- 77 adolescents in total: - 23 adolescents without varicocele; - 37 adolescents with varicocele and normal SA; - 17 adolescents with varicocele and abnormal SA.	- 541 proteins in total: - 108 overexpressed in adolescents without varicocele; - 26 overexpressed in adolescent with varicocele and normal SA; - 13 overexpressed in varicocele and abnormal SA.	- Candidate biomarkers: - Adolescents without varicocele: SDF4, LEFTY1, DNASE1 and PLPP1; - Adolescents with varicocele and normal SA: IGFBP7 and IGHG3; - Adolescents with varicocele and abnormal SA: CRISP3	- hexose metabolic process - secretory granule - wound healing - lysosome
Fariello et al. 2012 [60]	Smokers with varicocele	- 56 adult nonsmokers with varicocele; - 29 adult moderate smokers with varicocele; - 25 adult heavy smokers with varicocele.	- 20 identified proteins: - 4 proteins in nonsmokers - 5 proteins in moderate smokers - 1 protein in heavy smokers	- Nonsmokers: SERPINA1, ASAH1, CALM1, CRISP1; - Moderate smokers: ANXA3, CTSB, EDDM3B, PTGDS, SOD3; - Heavy smokers: AZGP1	- Lipid catabolic process - Inflammatory pathways - Proteolysis - Apoptosis
Antoniassi et al. 2016 [61]	Smokers	- 20 nonsmoking men; - 20 smoking men.	- 422 identified and quantified proteins: - 1 absent; - 6 over-represented; - 27 under-represented.	IGLC3, IGHA1, APOA1, S100A9, ORM1, LCN2 and SCGB2A1	- antigen processing and presentation - protein kinase A signaling and arachidonic acid secretion - complement activation - positive regulation of antigen processing and presentation - regulation of acute inflammatory response
Da Silva et al. 2013 [54]	Spinal Cord Injured	- 12 SCI men (6 collected by EEJ and 6 collected by PVS); - 10 healthy control donor.	- 637 identified proteins: - 35 exclusive in control group - 88 exclusive in PVS group - 66 exclusive in EEJ group	PVS proteins of interest: TRFL, CERU, C3, CPB, NFAC4, EP300, IGKC EEJ proteins of interest: SPTA2 and SYNE1	- cytoskeletal binding - response to calcium - oxidation of iron ion
Da Silva et al. 2016 [53]	Spinal Cord Injured	- 12 SCI men; - 11 healthy control donor.	- 2550 proteins identified in total: - 344 exclusive in SCI group; - 146 proteins exclusive in controls	PRTN3, ELANE, DEFA1, PZP and A2M	- inflammatory processes; - protease inhibition;
Camargo et al. 2015 [62]	Spinal Cord Injured	- 9 SCI men before and after oral treatment with probenecid	- 806 proteins in total: - 77 proteins over-expressed or exclusive pre-treatement - 33 exclusive or over-expressed after the treatment.	Pre-treatment: NEDD8, CUL3, PSMD7, PSMA8 and PMSB7. Post-treatment: GARS and GART.	- proteasomal degradation - ubiquitination - inflammatory activity
Milardi D. et al. 2014 [52]	Male Hypogonadism	- 10 men with secondary hypogonadism - 10 fertile and normogonadic men	- 61 proteins identified in fertile group - of which, 33 were absent in hypogonadic patients - 14 proteins found in hypogonadism men after therapy;	Therapy related proteins: S100A9, PIP, AZGP1, ACPP, LTF, MPO, CST3	- catalytic activity - binding activity - hydrolase activity

- *SA* semen analysis

- *SCI* spinal cord injured

Different studies have been performed in order to understand the intrinsic mechanisms of varicocele – the most prevalent cause of male infertility [53–57, 59–62]. In adolescents, seminal plasma proteomics analysis demonstrated that Cysteine-rich secretory protein 3 (CRISP-3) was highly expressed in adolescents with varicocele and with seminal alterations, 80-fold increased when compared to controls without varicocele or with varicocele and normal semen quality. In the future, this protein may potentially be used to increase sensibility in determining the best time to intervene in these adolescents. In that study, the authors were also able to demonstrate that varicocele shifts away the seminal plasma proteome from the profile presented by adolescents without varicocele, and that this is more intense in those adolescents with altered semen quality. The authors discussed that it seems that varicocele leads to an equilibrated, altered stated, different from a homeostatic state (a form of homeorrhesis) [57]. In a further confirmatory analysis of their results, the authors of then demonstrated that, in adolescents with varicocele, there is an increase in the seminal levels of IGFBP7 – a protein that participates in cell proliferation. Interestingly, only adolescents with varicocele who also presented altered semen analysis presented a decrease in the seminal levels of DNASEI – a protein involved in apoptosis [58]. The authors discussed that their results support the evidence that there is a general response to varicocele – an increase in cell proliferation – but that if apoptosis is decreased, the seminal phenotype is one associated to male infertility.

In adults with varicocele, seminal plasma proteomics studies demonstrated proteins linked to oxidative stress, and inflammation pathways. In addition, when the same patients were analyzed after varicocelectomy, proteins linked to energy production pathways and plasma membrane organization were increased. The authors discussed this is likely a shift back to homeostasis brought upon by intervention (in this case, surgical intervention), thus demonstrating that correction of varicoceles can alter the testicular environment – and that this is reflected in the seminal plasma proteome [55].

Because the male reproductive tract is very sensitive to environmental factors [63], the study of the seminal plasma proteome can help to observe early testicular alterations, even in the absence of alterations to semen quality [61]. In adult smokers, proteomics analysis revealed an inflammatory state of the accessory sex glands and testis, which in turn led to alterations to sperm DNA and acrosome integrity and to mitochondrial activity [61]. In addition, adult smokers with varicocele – in which there is a potentiation of the negative effects of smoking due to accumulation of toxins in the testes [64], Fariello et al. verified four exclusive proteins in moderate smokers related to apoptosis regulation, and that zinc-alpha-2-glycoprotein (ZA2G) protein was exclusive in heavy smokers with varicocele [60].

Another study focused on men with spinal cord injury (SCI). SCI leads to ejaculatory dysfunctions, ranging from retrograde ejaculation to anejaculation, and semen quality is characterized by very low sperm motility. Brackett et al. demonstrated that seminal plasma participates in determining this low motility. [53, 65]. Thus, da Silva et al. verified, using LC-MS/MS, that this impairment occurs due to an important prostate gland dysfunction added to increased immune system activity [53]. In another study, da Silva et al. also demonstrated that different assisted ejaculation techniques lead to different seminal plasma proteomic profiles. Using 2DGE and LC-MS/MS, the authors demonstrated that semen collected by penile vibratory stimulation (PVS) in men with SCI produced many exclusive cluster proteins involved in response to hydrogen peroxide and hypoxia, suggesting that ROS formation and oxidative processes are increased in these men [54]. Furthermore, in an intervention study, patients with SCI were treated with oral probenecid, which led to an increase in sperm motility was observed [66]. Subsequently, proteomics analysis was performed with the objective of understanding the molecular mechanisms that led to this increased motility. The authors demonstrated that, before the treatment, proteins enriched were linked to cell degradation, while after the treatment proteins enriched were linked to cell motility [62].

## Conclusion and final remarks

Seminal plasma is constituted of a mixture of secretions from accessory sex glands and from the testes, epididymides, and *vasa deferentia* [67]. When undertaking the study of the seminal plasma proteome, it is important to bear in mind that alterations in seminal plasma protein expression levels may arise from not only modulations to its expression per se (gene expression, mRNA translation, etc.), nor to its stability, but also from dilution of its fluid of origin by alteration in the relative contribution of one or another constituent. If, say, seminal vesicle contractibility is hindered due to alterations in smooth muscle cells that may arise from decreased testosterone levels [68], the relative contribution of prostatic and testicular/epididymal/vassal proteins will increase, but not because a true increase in expression level occurred. Understanding this effect is important when interpreting results from proteomics studies, and there is a need for the identification of proper normalizing proteins for each origin. It should also be mentioned that many studies of the seminal plasma proteome have encountered intracellular proteins. While it is not immediately clear as to why these proteins would be differentially expressed, some authors have suggested that the presence of dead or altered sperm in the male genitourinary tract would lead to the release of cellular constituents – among which proteins – into the epididymal/deferential fluid [8].

Current studies have demonstrated, however, that there is a homeostatic state of seminal plasma that effects sperm-related events (such as capacitation, energy production, and fertilization), and that cellular or biological conditions alterations shift this equilibrium away from this state. While many sperm-related functions are still observed in these samples, as would be expected (it is still seminal plasma), a number of different functions are observed that are not related to fertilization – almost as if seminal plasma loses its main focus on fertilization. The most commonly observed functional alteration is the characterization of an inflammatory state in semen. We have thus herein reviewed studies that have sought to demonstrate the seminal plasma proteome in male infertility, including a number of proteins suggested as markers for diagnosis and/or prognosis.

## Abbreviations

1DGE: One-dimensional gel electrophoresis
2DGE: Two-dimensional gel electrophoresis
iOAT: Idiophatic oligoasthenoteratozoospermic
IVF: In vitro fertilization
KEGG: Kyoto Encyclopedia of Genes and Genomes
LC MS/MS: Liquid chromatography mass spectrometry
MALDI-TOF: Matrix-assisted laser desorption/ionization time of flight mass spectrometry
MS: Mass spectrometry
NOA: Non-obstructive azoospermia
OA: Obstructive azoospermia
OAT: Oligoasthenoteratozoospermic
ROS: Reactive oxygen species
SCI: Spinal cord injury
SELDI-TOF-MS: Surface-enhanced laser desorption/ionization-time of flight mass spectrometry
